# Nursing students' experience of moral distress in clinical settings: A phenomenological study

**DOI:** 10.1002/nop2.2141

**Published:** 2024-03-15

**Authors:** Leila Mardanian Dehkordi, Toktam Kianian, Alireza Nikbakht Nasrabadi

**Affiliations:** ^1^ Nursing and Midwifery Care Research Center Isfahan University of Medical Sciences Isfahan Iran; ^2^ Nursing and Midwifery Care Research center Iran University of Medical Sciences Tehran Iran; ^3^ Department of Medical Surgical Nursing, School of Nursing and Midwifery Tehran University of Medical sciences Tehran Iran

**Keywords:** ethics, moral distress, nursing, phenomenology, qualitative research, students

## Abstract

**Aim:**

To explore nursing students' moral distress (MD) experiences in clinical settings.

**Design:**

An interpretative phenomenological analysis (IPA) design was employed.

**Methods:**

Purposive sampling was used. In‐depth semi‐structured face‐to‐face interviews were conducted from December 2020 to June 2021 with nursing students who were taking the internship course in clinical settings. Data analysis was conducted following Dickman et al.'s (1989) method.

**Results:**

Ten nursing students participated in this study. Three main themes were identified, including (1) negative learning environments, (2) internal disgust and (3) threats to professional identity.

**Conclusion:**

Findings showed that value conflict, lack of knowledge of ethical standards and its application, and unprofessional approaches result in negative environmental learning perceptions from the nursing students. Therefore, due to being unable to change the situation, they start to feel guilt and shame and, as a result, decide to escape the problem instead of managing it. These feelings lead to internal disgust. This issue indicates the importance of improving the knowledge and perception of these situations. Thus, nursing students must be prepared for the real world, where their ideals are constantly challenged. MDs were experienced as threats to dignity, inequality, distrust, and change of mentality towards nursing, characterised as threats to professional identity. It is suggested to inquire about the process of nursing students' resiliency in morally disturbing situations to deduce the suitable approach for clinical education.

## 1 INTRODUCTION

One of the integral components of the nursing profession is ethical decision‐making based on moral beliefs (Saeedi et al., 2019; Shirazi & Sabetsarvestani, 2021). In this profession, individuals are challenged with ethical questions (Deschenes et al., 2020) that can lead to moral distress (MD). According to Jameton and Wilkinson, MD is the emotional disturbance and negative emotional state experienced by nurses when barriers in the clinical environment prevent them from acting in line with their moral judgements (Jameton, 1984; Wilkinson, 1987). (Appendix S1)

Among nursing students, MD is a common problem that could contribute to a critical shortage of nurses in the future (Baghdadi et al., 2020; Bordignon et al., 2019; Janatolmakan et al., 2021). Nursing students experience MDs throughout different phases of their clinical training process. They often encounter MDs in conflict or dilemma regarding ethical–moral values. In such conditions, students feel urged to act but are unable to, reflecting the motivation to perform healthcare practices based on ethical dimensions (Rennó et al., 2018). Mazzotta et al. reported that MDs are instilled in students derived from five aspects of improper institutional situations to teach user care, authoritarian teaching practices, disrespect for the ethical dimension of vocational education, and lack of teacher competence and commitment to the ethical dimension of user care (Mazzotta et al., 2022).

## 2 BACKGROUND

The moral problems experienced by nursing students differ from those experienced by nurses, and the actions they take are different as well (Shayestehfard et al., 2020). They find themselves highly responsible for their patients (Bordignon et al., 2019); also, they feel MD in cases where they cannot take the right action due to various reasons (e.g. social pressure to conform with others, limited opportunities to talk over moral issues, restrictive power structures, and no academic support and organisational priorities) (Lamiani et al., 2017; Wojtowicz et al., 2014). The most MD‐related issues among nursing students are insufficient competence, poor quality of patient care, inadequate communication and cooperation of health care providers with students, which are described as morally disturbing situations in practice (Escolar Chua & Magpantay, 2019; Escolar‐Chua, 2018; Theobald, 2013).

Concerningly, if a student has not received professional training, he will face many moral distresses (Cynthia Clark, 2017), and as a result, they are susceptible to mental and physical health problems (Lerkiatbundit & Borry, 2009; Pijl‐Zieber et al., 2008). In addition, a lack of MD assessment can lead to moral residue (Epstein & Hamric, 2009), reduce students' sensitivity to this issue, and eventually will make it a routine and repetitive problem (Bordignon et al., 2018).

Because of the adverse effects of MD, it is necessary to assess individuals' experiences to understand both the phenomenon and the context in which MD occurs (Morley et al., 2019). Primarily because of the personalised sociocultural construct of MD (Sasso et al., 2016) and different social and cultural backgrounds that can lead to different ethical perceptions and religious understandings and consequently affect their MD (Bordignon et al., 2019; Sasso et al., 2016). MD in undergraduate nursing students was evaluated in different contexts (Sasso et al., 2016), but there is limited information about nursing students' experiences in Iran.

Iran has an Islamic culture, and Iran's healthcare system is based on religious and cultural views (Al‐Ansari et al., 2020). Also, Iran is characterised by ethnic diversity (Karimi & Moazzen, 2011), which may affect nursing students' sociocultural backgrounds, moral and religious knowledge, and perceived MD.

Therefore, given the significant impact of MD on future nurses' profession and the importance of its evaluation among various cultural contexts and populations, this study aimed to explore nursing students' lived experiences of MD in clinical settings. Additionally, it is aimed to explore an understudied phenomenon and highlight the need for problematisation in nursing education of the causes and outcomes of MDs for professional socialisation.

This study can ease MD comprehension to help educational institutions monitor the status of nursing students, develop coping and intervention strategies to maintain students' well‐being, and ensure their quality of education.

Research question: What are nursing students' lived experiences of MD in clinical settings?

## 3 THE STUDY

### 3.1 Aim and objective

The current study aimed to explore nursing students' MD experiences in clinical settings.

## 4 METHODS

### 4.1 Design

An interpretative phenomenological analysis (IPA) design was employed.

### 4.2 Theoretical framework

The current study employed an interpretive phenomenological design to explore the lived MD experiences of nursing students in clinical settings. Descriptive phenomenological research is based on the belief that researchers should immerse themselves in the phenomena they study. This design allows researchers to understand better each participant's experiences (Speziale et al., 2011). According to a constructivist paradigm, phenomenological research is oriented by a relativist ontology in which human realities are conceived as multiple, intangible mental constructions based on social and experimental information that are local and particular. Researchers use this paradigm to emphasise the contextual nature of qualitative research findings, which are elicited through co‐construction with participants.

In contrast to other qualitative research traditions, interpretive phenomenology is grounded in a unique understanding of being' (Frechette et al., 2020). Phenomenology helps us study the lived experience and the people as they are experienced (Moustakas, 1994). A lived experience method was used in this study due to the fact that nursing students provided their perspectives on MDs' explanations.

### 4.3 Participants and recruitment

The current study samples were nursing students who were taking the internship course in the teaching hospitals of Tehran University of Medical Sciences. There are 130 units in the Iranian BSN program which includes 22 general, 15 basic sciences, 54 core, 18 clinical training and 21 units. Iran's clinical training process is arranged from simple to complex and takes place during a patient's care based on the nursing process. Students' clinical training starts in the second semester and will run till the end of the third year, concurrently with their theoretical courses. During the fourth year, students undertake a full‐time apprenticeship in a hospital. This course is called an internship course which students can work with patients in various departments of general hospitals (e.g. internal‐surgical, paediatric, obstetrics, gynaecology, psychiatry, emergency and critical care) (Farsi et al., 2022). In this study, nursing students who met the following criteria were eligible to participate: (1) undergraduate students who entered the internship course, (2) successfully completed all their theory courses and (3) have experienced MD. No exclusion criteria were set in the current study. The study employed a purposive sampling method. Participants who tended to take part in the study were recruited from different wards during their clinical education.

### 4.4 Data collection

Once the consent form is signed. The interviews were conducted in a quiet place in the hospital. Anonymity and confidentiality of the information were maintained. Data were collected through in‐depth semi‐structured face‐to‐face interviews between December 2020 and June 2021.

An interview guide was developed based on the primary interview. Every interview started with open questions. The interview questions included the following: (1) what comes to your mind when I say MD? (2) How do you describe MD? (3) How do you feel when MD occurs? (4) Can you give an example? Probing questions, such as ‘Can you explain more? Have you had any similar experiences?’ were used to gather more details on the experiences. In order to ensure consistency, all interviews were conducted by LM, a female PhD in nursing with experience in phenomenological research.

The interviewer did not know any of the participants before the study. The interviews began with open‐ended questions followed by self‐introductions. During the interview, participants were encouraged to talk freely about their experiences. The interviews were conducted in a quiet room, audio‐recorded and lasted 60–120 min. Field notes were made by LM for each interview. Ultimately, the participants were asked to comment if anything was left to say. Interviews continued until reaching depth, richness, abstraction and relevance.

### 4.5 Data analysis

Verbatim transcriptions of audiotape interviews have been generated. Three researchers (LM, AN and TK) analysed the data in order to reduce the researchers' personal experiences and beliefs influence. The seven‐step phenomenological data analysis method was followed (Diekelmann et al., 1989). (1) The researcher listened to the interview recordings several times. The recordings were then transcribed and reviewed to obtain a general understanding. After that, the transcripts were returned to the participants to check if there were any misunderstandings or mistakes; (2) Interpretative abstract for each interview was written by the researcher, and the underlying themes emerged. Afterwards, the extracted themes were included in the relevant categories; (3) Research team members discussed the extracted themes once more and shared their ideas to reach a consensus over the emerged themes. The subsequent interviews complemented the previous themes obtained during earlier analysis and, sometimes, led to new emerged themes. Interpretations and patterns were developed while finalising the themes; (4) The researchers frequently reviewed the transcripts for clarification, outlining a new classification of the extracted themes and resolving any inconsistencies among research team members and/or their interpretations; (5) Recurring themes in the transcript that disclosed participants' shared experiences of MD were identified; (6) The transcripts were compared, and the themes were discussed in terms of interrelationship; (7) A ‘constitutive pattern’ was formulated, and its final draft was delivered to the interpretation group to be examined (Diekelmann et al., 1989). Data were analysed manually.

### 4.6 Ethical considerations

This research project was approved by the Ethics Committee of REDACTED. Written informed consent was obtained from the participants regarding the aim of the study, confidentiality of the information, anonymity of the data, voluntary participation in the study and that nonparticipation would not lead to any disadvantages for them. They could leave the study at any stage they desired. The study followed the Declaration of Helsinki.

### 4.7 Rigour

To determine the study's rigour, the criteria of Lincon and Gaba (1986) were used to establish the study's credibility, dependability, conformability and transferability (Lincoln & Guba, 1986). Data credibility was achieved through close interaction with participants, long‐term and continuous engagement with the data and the application of a team approach throughout the stages of the study. The interviews were transcribed by the first author instantly after the end of each interview. The analysis of the data was done in a stepwise manner, following the hermeneutic circle. All evidence and documents have been saved safely to maintain dependability (repeatability). Also, the findings from the participants were presented using thick descriptions, the context was defined in sufficient detail, and quotes from the participants were used to allow the reader to make a transferability judgement (Lincoln & Guba, 1986).

## 5 FINDINGS

### 5.1 Participants

The study included six female and four male nursing students with different marital statuses (three married and seven single). The average age of the participants was 22.4 ± 0.51 years.

Three themes were identified during the analysis of the interviews, which included the following: (1) negative learning environments, (2) internal disgust and (3) threats to professional identity (Table 1).

**TABLE 1:** Sub‐theme and themes.

Themes	Sub‐themes
Negative learning environments	Value conflict
	Weaknesses in the knowledge of ethics and its application
	Unprofessional approaches
Internal disgust	Sense of guilt and shame
	Escaping the situation
Threats to Professional Identity	Threats to dignity
	Inequality
	Distrust
	Change of mentality towards nursing

### 5.2 Theme 1: Negative learning environments

The negative learning environment theme shows that the environment in which students should feel safe and supported in seeking knowledge and skills has turned into a distressing and discouraging environment due to experiencing value conflict, weaknesses in the knowledge of ethics and its application, and unprofessional approaches.

#### 5.2.1 Value conflict

During the clinical training course, students frequently encountered events contrary to moral values. The moral conflicts the students have repeatedly experienced in wards have made them discouraged, confused, scared and disappointed. One student described his experiences of the conflict between some nurses' behaviour and moral values as abandoning ethics and values, as well as being far from humanity:

It looks like a person has learned a few procedures and abandoned any moralities. Such person can't be called a nurse. She/he routinely does some procedures until retirement without adhering to ethical principles. These conflict with our beliefs. It's sad and far from humanity. I'm ashamed that my colleague…. (Participant No. 1).

Another student says that there is a conflict between our religious values and existing conditions:

We had a young female patient in the ICU who was in the end stage and unconscious. A young male came for help to change her clothes. He didn't consider the dignity of the patient and the principles of changing clothes and sheets. He unclothed the patient in front of us and our male classmates. (Participant no. 6)

Another student experienced confusion due to the conflict between what he has theoretically learned and what is happening in the clinical training settings:

In the clinical settings, you experience many situations different from what we have learned in the past few years as moral education, but no one follows them in practice. Finally, one wonders what to do. (Participant no. 6)

#### 5.2.2 Weaknesses in the knowledge of ethics and its application

Students had experienced limited knowledge of ethics and its application at different levels. Based on their experience, some instructors with sufficient scientific and clinical expertise lack knowledge of medical ethics. In addition, according to their experience, in many cases, the staff either do not have adequate ethical knowledge or, if they have any, ignore the principles in practice.

One of the students mentioned the instructors' weaknesses in this area:

We never had an instructor who would explain to us and clarify what we have learned in the classroom regarding a clinical setting. Instructors usually don't care about it, and they often pass these things easily. (Participant no. 5)

The students have repeatedly experienced the staff's unethical emphasis and guidance regarding ethical principles. In this regard, a student noted that:

There was a patient for whom the care measures were not completely done for. When I informed the ward personnel, he said, don't involve yourself so much with the patient; nothing will happen. (Participant no. 4)

#### 5.2.3 Unprofessional approaches

The students had experienced the staff and even the instructors' unprofessional and unethical approaches, which had negatively affected their learning environment.

Students complained about the wrong and unsafe procedures the staff performed and their inappropriate behaviour towards the students. They also complained that they ignored the students.

I saw that the nurse was not properly suctioning the patient's airway; he also didn't follow the sterile principles. I told him that he was doing it wrongly. He didn't pay any attention to this issue and continued the procedure in a lousy mood while mumbling under his breath. We have seen these things a lot. (Participant no. 5)

The staff's misbehaving and disregarding patients' physical and mental conditions annoyed and distressed the students. One of the interviewees stated:

There was a patient who hadn't bathed for three months and had a mental disorder. He also had delusions. He told the nurses, “you can not force me to take a bath,” but the nurses pulled him to the bathroom on the ground in front of others. The blood overflowed the catheter. His slippers were dropped off his feet. It was really a terrible scene. (Participant no. 4)

Participants also reported the problems of student–instructor relationships. Some interviewees mentioned that some instructors' unethical behaviour is primarily attributed to their personality, which sounds like they have not been taught ethics.

I think someone who misbehaves in the ward is not a professional person. For example, if a nursing instructor humiliates a student in front of a few instructors, nurses, or even the rest of the students, is not worthy of being an instructor. When he comes to the ward, he humiliates you the same way, and this behaviour is repeated everywhere. (Participant no. 6)

Some participants had experienced some health workers' irresponsibility towards patients, which not only lowered the quality of care and treatment but also threatened the patients' life.

One of the doctor's orders for the patient was carried out, and he became agitated, and his condition worsened. Then to hide this medical malpractice, the doctor added another order between the last order and the line drawn by the nurse. He blamed it on the poor nurse, insisting that I had written the order and this woman who did not follow it, is responsible. Well, it was awful. (Participant no. 4)

### 5.3 Theme 2: Internal disgust

The feeling of internal disgust was another theme that the students experienced due to senses of shame, guilt and the desire to escape from the situation when facing MD.

#### 5.3.1 Sense of guilt and shame

Some feelings, such as discomfort and regret, originated from the students' inability to face unethical behaviour and made them upset. When nursing students witness the staff's unethical behaviour, they imagine that 1 day their dear ones and they could also be in the same situation, and this makes them feel upset:

Once, there was a nurse who was brutally suctioning the patient. Whenever I remember that scene, I feel upset. I wonder why I couldn't stop her and tell her that you are wounding the entire lung, and I really hate myself. (Participant no. 5)

On the other hand, MD was experienced as feelings of shame and mostly embarrassment, demoralisation and inability to forget. An example of these feelings is as follows:

Once, there was a family caregiver who was not very alert. The nurse spoke to him insultingly, and he obeyed desperately. I felt ashamed and embarrassed to be there at that time and situation, where I couldn't help, and I just witnessed my colleague's disgusting behaviour. (Participant no. 8)

#### 5.3.2 Escaping the situation

Perceived MD in the clinical education setting has made the students leave the situation and escape from the perceived tensions. One of the students who requested to change her ward said:

When this happened to me, I called our instructor to come and change my ward, and he changed it. The atmosphere there was really bothering me. (Participant no. 1)

The students found themselves in a situation where they could not do anything to relieve their MD, and they had no choice but to remain silent. It was an unpleasant condition that they wished to end:

When I see these unethical behaviours, I wish my college period ends soon… When you are a student, you don't have the power to do anything; you must be silent and just tolerate the situation. (Participant no. 7)

### 5.4 Theme 3: Threats to professional identity

One of the most important themes of this study was the threat to professional identity in nursing students. This identity they have shaped via the university educational system is threatened by experiences such as threats to dignity, a sense of inequality, and a change of mentality towards nursing.

#### 5.4.1 Threats to dignity

Students not only experienced disrespect for the patient's dignity but were also threatened in clinical settings. Hence, one of the sub‐themes that led to the deterioration of the professional image was the threat to students. Students repeatedly mentioned experiences such as being abused, humiliated, forced to do what they did not desire and feeling crushed in clinical settings. One of the students told us about the experience of being humiliated:

Our instructor asked us a question to which none of us knew the answer. Then he started talking to us in a very, very ridiculous tone. He told us to throw ourselves out of the window one by one, and he continued to say some sarcastic words. They treated us so badly that we no longer dared to go over that patient's bed. (Participant no. 3)

#### 5.4.2 Inequality

Another sub‐theme reflected the deterioration of the professional image and the sense of inequality perceived by the students. Some students talked about inter‐professional discrimination:

It is very sad that I, a senior nursing student, cannot sit at the nursing station, but an intern can, or they pull the medical record out of a nursing student's hands. You never see such behaviour towards a student of medicine. (Participant no. 1)

It has happened many times that we were inside the conference room to report a case, but in the middle of the program, they interrupted us and said to go outside. They want us to obey because, for example, medicine students have a class. I think this behaviour is not fair and appropriate. (Participant no. 9)

#### 5.4.3 Distrust

The lack of trust in students' clinical competence and ability to do the work was another sub‐theme contributing to the deterioration of the students' professional image. In this regard, one of the participants shared his experience with us and said:

As soon as I wanted to adjust his serum drops, he didn't allow me to do that. He yelled at me and said don't touch me; you don't know anything; I was about to cry; I tried not to react; I just left the room. (Participant no. 5)

#### 5.4.4 Change of mentality towards nursing

Finally, another sub‐theme that reflected the deterioration of the professional image was the change of mentality towards nursing. This feeling was developed due to the moral conflicts in nurses, instructors and peers' behaviour. One of the students expresses his feelings about nurses' unethical behaviour as follows:

When patients encounter such nurses (i.e. those who misbehave), they generalize and think all nurses are like this. I also sometimes say that most nurses are really like this (i.e., they misbehave.), and I regret that. (Participant no. 2)

Two other students admitted that:

Sometimes you see some behaviours that entirely change your view of the field. Seeing such behaviours makes you lose interest in the field and the job. (Participant no. 5)

Behaving this way changes everyone's view of nursing. Public sees nurses as bad‐tempered. Of course, not all nurses are like this, but they see those few bad‐tempered nurses and generalize it to all. (Participant no. 3)

## 6 DISCUSSION

The current study explored the experiences of nursing students' MDs in clinical settings. The nursing students experienced negative learning environments, internal disgust and threats to professional identity in clinical settings. The study findings provide valuable information for educational organisations to build a safe and positive setting to support nursing students and help them manage MD.

Negative learning environments were perceived as MD by the students. The findings of this article are consistent with the results of different authors, which determined that nursing students experience periods of MDs in their learning environment and clinical course due to value conflict, weaknesses in the knowledge of ethics and its application and unprofessional approaches (Escolar Chua & Magpantay, 2019; Sasso et al., 2016). The reason for this finding may be the transition of nursing students from an ideal education in the controlled environment of the college to the real world and eventually experiencing morally disturbing situations that they cannot manage (Bordignon et al., 2018). Zuzelo stated that current nursing education programs do not adequately teach students how to identify their feelings about MD, much less how to deal with them (Zuzelo, 2007). Consistent with the results of the present study, Rennó et al. reported that nursing students had experienced MDs at all stages of the nursing education process, especially in clinical and professional training. As a result, MDs caused by education/curriculum and location contribute to students' negative learning environment. In this study, nursing students cited misuse of techniques, medical dominance, and deficiencies in health services as sources of MDs (Rennó et al., 2018). In this regard, Janatolmakan et al. pointed out that nursing students are involved in value conflict, weaknesses of moral knowledge and unprofessional approaches; for this reason, they report the learning environment as negative and unethical (Janatolmakan et al., 2021; Reader, 2015). Another study also revealed that nursing students face MDs related to perceived unethical and unprofessional behaviour of others in the clinical setting (Escolar Chua & Magpantay, 2019). Sasso et al. also pointed out that health care disparities and inequalities, ethnic origin, religious faith, individual characteristics of students, their values, and lack of awareness and sensitivity to patient's rights and ethical aspects can all have a negative impact on the decisions taken and the nursing care provided, eventually creating MDs (Sasso et al., 2016). Matchett and Stanley also emphasised that personal conflict as a result of differing value systems may be a moral challenge faced by nursing students in clinical settings (Matchett & Stanley, 2014). Altogether, this may show that the training provided in the classroom does not prepare nursing students to face morally disturbing situations in the real world, and clinical educators do not have the necessary competence to support students. Educators should use supportive strategies; for example, they must explain to nursing students whenever they face distress, discuss the issue and ask them about the values and principles they have learned, and help them to be morally sensitive so that students can deal with various ethical conflicts that may arise in the clinical settings (Escolar Chua & Magpantay, 2019).

This study found that nursing students experienced feelings of guilt and shame and escaped from the situation in clinical settings, which conveyed MD to them by creating a sense of internal disgust. In this regard, Nasrabadi et al. (2018) also reported that when people act against their interests and beliefs, they experience MD and are subsequently judged morally. They constantly blame themselves for knowingly committing an immoral act, and as a result, they feel shameful, which makes them feel disappointed and emotionally distressed (Nasrabadi et al., 2018). Vittone and Sotomayor (2021) also emphasised that MD can be expressed in the form of painful emotions and feelings and also in the form of psychological pain and suffering (Vittone & Sotomayor, 2021). Similarly, Willis (2015) acknowledged that morally distressed people report anger, frustration, resentment, sadness, anxiety, shame, embarrassment and disgust (Willis, 2015). According to Wijma et al. (2016), silence and shame caused by MD are signs of moral responsibility in an individual, which has been neglected for some reasons (Wijma et al., 2016). To prevent moral conflict, students rely on emotional distancing (Matchett & Stanley, 2014) or due to the fear of facing adverse psycho‐social conditions and losing the practical learning opportunity, they remain silent and even avoid challenging clinical situations (Baraz et al., 2015) Escolar‐Chua also reported that, most nursing students ignore morally distressing situations to avoid conflict and confrontation (Escolar‐Chua, 2018). In this regard, Bordignon et al. (2018) stated that when students cannot change a situation based on what they have learned, they suffer and prefer to change their clinical education ward (Bordignon et al., 2018) In relation to Matchett and colleague also expressed that when faced with moral distress, nursing students relied on the strategy of distancing themselves from difficult emotions instead of trying to resolve their internal conflict (Matchett & Stanley, 2014). So, a deep understanding of how MD occurs, its characteristic signs and symptoms, and which educational factors and experiences can have the strongest impact on students can help develop and test preventive strategies (Sasso et al., 2016).

The threat to professional identity was another important theme of this study, which was comprehended through experiences such as threats to dignity, a sense of inequality, distrust and a change of mentality towards nursing. The results of the study conducted by Reader (2015) align with the present study's findings in which nursing students experience feelings such as inequality, blame and intimidation in the transition from college to professional practice. They report learning and working in an unjust culture and suffering from it (Reader, 2015). Consistent with the results of the present study, in the study of Rennó et al., nursing students experienced MDs in the form of being ridiculed and disrespected by others, especially the teacher. They believed that a teacher who lacks an ethical attitude could not provide ethical training (Rennó et al., 2018). Clark (2013) asked nursing instructors to review their interactions, model respectful behaviour and promote it among their colleagues in the wards. Modelling professional behaviours is considered one of the most effective tools for teaching professionalism (CM Clark, 2013). When students observe unethical incidents and behaviours, they lead to ‘traumatic de‐idealisation’ (Fayez et al., 2013). These negative impacts not only influence students' behaviour, confidence and attitude but also have destructive effects on the social and professional consequences of nursing (Baraz et al., 2015; Bhurtun et al., 2019). In this regard, Wojtowicz et al. also reported that experiencing MD can lead nursing students to leave the workplace just to maintain their moral integrity and prevent burnout (Wojtowicz et al., 2014). Also, Scalarcho emphasised that it is essential for nursing students to recognise distressful situations and learn how to deal with them because negative experiences and environments can be an important deterrent to their staying in the nursing profession (Escolar‐Chua, 2018). Therefore, developing an environment accompanied by respect and civility in clinical training is vital to maintain a suitable professional image for students.

### 6.1 Limitations of the study

This study has done in two sex groups (male and female), and the participants were students who worked in different clinical wards (critical and general wards). If it had been done only in one sex group or one clinical ward, the result could've been different.

### 6.2 Recommendations for further research

The qualitative study provides an in‐depth understanding of students' experiences of MD in the clinical setting. Moreover, these findings can be used to improve the education of future nurses by the education and healthcare systems. In order to improve the quality of education and psychological and scientific support of students by their peers (higher level students), instructor, and professor, a sequential training plan is recommended to identify and correct scientific and functional defects. When this model is implemented, fewer questions and challenges will remain unanswered, and the academic and functional deficiencies of the trainer and even clinical personnel will be eliminated. Moreover, care patterns and weaknesses are corrected, and the skills to deal with moral distress are learned instantly. The student will then not feel abandoned in an environment accompanied by distress. Also, professionals can fill the gap between theory and practice by responding quickly and accurately to distresses that are present at the moment.

It is suggested to inquire about the resiliency of nursing students in morally disturbing situations in order to revise and review existing clinical education approaches.

## 7 CONCLUSION

Iranian students experienced MDs by understanding the negative learning environment, internal disgust and threat to professional identity in clinical settings. Findings indicate the need to use ethical strategies to minimise distress and pay more attention to ethics, knowledge, and educational skills in clinical settings. To this end, it is suggested that the internship plan ‘aimed at empowering students (future nurses)’ be revised by including an ethical training program. Implementing an ethical training program by competent trainers can enable students to manage the MD felt in the clinical setting.

## AUTHOR CONTRIBUTIONS

Study conception and design, data collection and data analysis, manuscript drafting: LM; data analysis and manuscript drafting: TK; data analysis and manuscript critical revision: AN. All the authors have read, provided feedback and approved the final manuscript.

## FUNDING INFORMATION

This study was supported by Tehran University of Medical Sciences (IR.TUMS.VCR.REC.1397.567). Funders played no role in the conceptualisation, design, drafting and approval of the manuscript.

## CONFLICT OF INTEREST STATEMENT

The authors declared no conflicts of interest regarding article publication.

## CONSENT FOR PUBLICATION

Not applicable.

## RESEARCH ETHICS COMMITTEE APPROVAL

The present study received approval from the Ethics Committee of the Tehran University of Medical Sciences, Tehran, Iran, with the code: (IR.TUMS.VCR.REC.1397.567). The Declaration of Helsinki was considered in this study. Written informed consent were obtained from participants. They received written information regarding the purpose of the study, confidentiality of the information, anonymity of the data, voluntary participation in the study, and that nonparticipation would not lead to any disadvantages for them. They have the right to withdraw from the study anytime they wish. Also, the Committee on Publication Ethics was considered.

## Supporting information


Appendix S1

## Data Availability

The datasets used and analysed during the present study are available from the corresponding author upon reasonable request.
